# Voluntary exercise improves spermatogenesis and testicular apoptosis in type 2 diabetic rats through alteration in oxidative stress and mir-34a/SIRT1/p53 pathway

**DOI:** 10.22038/ijbms.2020.49498

**Published:** 2021-01

**Authors:** Saber Gaderpour, Rafighe Ghiasi, Golamreza Hamidian, Hamed Heydari, Rana Keyhanmanesh

**Affiliations:** 1 Department of Physiology, Faculty of Medicine, Tabriz University of Medical Sciences, Tabriz, Iran; 2 Drug Applied Research Center, Tabriz University of Medical Sciences, Tabriz, Iran; 3 Department of Basic Sciences, Faculty of Veterinary Medicine, University of Tabriz, Tabriz, Iran; 4 Neurogenic Inflammation Research Center, Mashhad University of Medical Sciences, Mashhad, Iran

**Keywords:** Apoptosis, miR-34a, Oxidative stress, p53, Sperm parameters, Type 2 diabetes, Voluntary exercise

## Abstract

**Objective(s):**

This research was designed to demonstrate the impact of voluntary exercise on sperm parameters including sperm count, morphology, motility, viability, testicular apoptosis, oxidative stress, and the mir-34a/SIRT1/p53 pathway in type 2 diabetic rats.

**Materials and Methods:**

32 Wistar male rats were separated into four groups: control (C), voluntary exercise (VE), diabetic (D), and diabetic rats that performed voluntary exercise (VED). To induce diabetes, animals were injected with streptozotocin (35 mg/kg) after receiving a high-fat diet. The testicular protein levels of SIRT1 and P53, miR-34a expression, MDA, GPx, SOD, catalase, and sperm parameters were evaluated.

**Results:**

Diabetes caused increased testicular MDA content, miR-34a expression, acetylated p53 protein expression, and the percent of immotile sperm (*P*<0.01 to *P*<0.001) as well as reduced testicular GPx, SOD and catalase activities, SIRT1 protein expression, and sperm parameters (*P*<0.05 to *P*<0.001). Voluntary exercise reduced testicular MDA content, miR-34a, and acetylated p53 protein expression compared with the D group (*P*<0.001), however, GPx, SOD, catalase activities, and sperm parameters in voluntarily exercised rats were elevated compared with diabetic rats (*P*<0.05 to *P*<0.001).

**Conclusion:**

It seems that voluntary exercise has significant positive impacts that can be employed to reduce the complications of type 2 diabetes in the testis of male rats.

## Introduction

One of the major universal metabolic disorders worldwide is diabetes, which causes reduced fertility (1, 2). Nowadays diabetes-related reproductive disorder is a new and urgent challenge because of its high prevalence in younger people (3). At several levels, diabetes may affect reproductive functions in the testis including changes in sperm quality, spermatogenesis, and testosterone depletion (3). In diabetic males, numerous studies have shown changes in sperm motility, count, and morphology (4-6), but the results are inconsistent and some studies have failed to identify such spermogram pathologies (4, 7, 8). Diabetes also reduces spermatogenic cells and causes seminiferous tubular atrophy. These changes reflect morphological abnormalities in spermatogenesis (9, 10). Diabetes mellitus can cause reproductive complications through various mechanisms, including oxidative stress and apoptosis.

An important risk factor for developing diabetic complications is oxidative stress (11, 12). Increased free radical levels and simultaneous reduction of antioxidant defense mechanisms lead to elevated lipid peroxidation, insulin resistance, and damaged enzymes and cellular organelles. These outcomes of oxidative stress may cause an increase in diabetic complications (13). Silent information regulator 1 (SIRT1) is a potential target of miR-34a (14) which has a crucial function in regulating the cell cycle, metabolism, and oxidative stress-induced apoptosis (15). Growing data have shown the importance of miRNAs as a new aspect in the regulation of spermatogenesis and finally male fertility (16). One of the miR-34 family members is miRNA-34a and it is extremely expressed in the testicles (17). MiR-34a is an oxidative stress-responsive RNA that deals with deleterious conditions (16). High oxidative stress can trigger the apoptosis of testicular tissue, eventually resulting in infertility (18). A study showed that mir-34a performs a crucial part in spermatogenesis and spermatozoa function (16). SIRT1 may be negatively regulated by miR-34a; it can directly bind to SIRT1 mRNA and control cell apoptosis in a cell culture model by repressing SIRT1 (19). Chang* et al.* (20) suggested that miR-34a is crucial in the p53 tumor repressor system; the activation of miR-34a-dependent on p53 is broadly confirmed and its up-regulation leads to cell cycle arrest and apoptosis. The relation between miR-34a and p53 is complex; the major commonly diagnosed pathway is the SIRT1-dependent pathway (14, 21-23). The researchers confirmed that miR-34a is elevated in diabetes and miR-34a knockdown inhibits pancreatic β cell apoptosis and thus preserves the number of β cells (24, 25).

Currently, several medications for lowering blood glucose levels are utilized to manage and treat diabetes, which can have negative impacts on different organs. An investigation showed that using sulfonylureas can lead to beta-cell apoptosis and long-term treatment failure (26). Moreover, researchers reported that glibenclamide and metformin can reduce the antioxidant status of testicles, which leads to testicular impairment and diminished sperm count and motility (27). 

To reduce these side effects, researchers have tried to employ alternative treatments for controlling diabetic complications. Several studies on physical exercise training, firmly support its effectiveness to prevent and manage diabetes (28). Exercise improves insulin sensitivity in individuals and reduces the risk of developing type 2 diabetes mellitus in animals (29, 30). Since forced-exercise models are stressful, they may be problematic, which implies that voluntary exercise can be a more useful model (31). So, this research is proposed to investigate the impact of voluntary exercise in male type 2 diabetic rats on sperm parameters, testicular tissue oxidative stress, apoptosis, and expression levels of SIRT1, P53, and mir-34a.

## Materials and Methods

***Animals and study design***

Thirty-two Wistar male rats (200–220 g) were acquired from the Laboratory Animal House of Tabriz University of Medical Sciences. Rats were kept in standard circumstances (lights on from 8.00 am to 8.00 pm , 22±2 °C) and had open access to food and fresh water for 15 weeks. All of the procedures were authorized by the Animal Care Committee of Tabriz University of Medical Sciences (IR.TBZMED.VCR.REC.1397.127). Rats were randomly separated into four groups (n=8): healthy control (C), voluntary exercise (VE), diabetic (D), and diabetic rats treated by voluntary exercise (VED).

***Induction of type 2 diabetes***

Rats in D and VED groups received a high-fat diet (HFD) regimen containing 48% carbohydrates, 20% protein, and 22% fat for 4 weeks (31). After the dietary regime, a low dose of streptozotocin (35 mg/kg, Sigma-Aldrich, St. Louis, MO, USA) was injected intraperitoneally (32). Five days later, rats with fasting glucose of greater than 250 mg/dl were regarded as diabetic (33).

***Voluntary exercise***

In the voluntary exercise group, animals were separately kept in a stainless-steel cage (Tajhiz Gostar, Tehran, Iran) and permitted to have open access to the wheel 24 hr a day for 10 weeks. Animals were exercised based on their physiological threshold for normal activity. This voluntary exercise was acknowledged as a mild-to-moderate exercise (34). The running distance was monitored daily by a sensor placed on each running wheel. Animals with running intervals less than ~2000 meters per 24 hr were omitted before statistical analysis (28). Furthermore, daily running distances of rats in the VED group were assessed separately for 10 weeks after diabetes confirmation. The sedentary animals were maintained in regular cages for equal time without running wheels.

***Anesthesia method and tissue sampling***

All rats were anesthetized after 15 weeks by intraperitoneal injection of ketamine (50 mg/kg) and xylazine (5 mg/kg). After sacrificing the rats, left testicles and epididymis were immediately removed and washed with cold saline normal and kept at -80 °C for determination of SIRT1 and P53 protein levels, miR-34a expression level, and oxidative stress. The right testicles were fixed in a neutral formalin buffer (10%) for apoptosis analysis. Finally, animals were euthanized by decapitation. 

***Western blotting***

The upper part of the frozen left testis was homogenized in ice-cold RIPA lysis buffer and centrifuged at 14,000rpm for 10 min at 4 °C. Supernatants were assembled, kept at −80 °C, and finally, the concentration of proteins was calculated via Bradford’s procedure. The cell lysates (50 µg protein/lane), isolated via sodium dodecyl sulfate-polyacrylamide gel electrophoresis (SDS-PAGE) were loaded and transferred on the polyvinylidenedifluoride (PVDF) membranes (Millipore, Billerica, MA, USA). The membranes were incubated via primitive antibodies overnight at 4 °C with Goat Anti-Rabbit IgG (H+L) Cross-Adsorbed Secondary Antibody, Alexa Fluor 594 (R37117) and immersed in ECL Plus Western Blotting detection reagent and displayed on Hyperfilm ECL (both from Amersham, Piscataway, NJ, USA). The band’s intensity was calculated using Lab Works 4.5 software (UVP, Upland, CA, USA). The primary antibodies used for Western blotting were β-actin (sc-47778), Sirt1 (sc-74465) (Santa Cruz Biotechnology, Inc.), and Acetyl-p53 (Lys382) Antibody (Cell Signaling Technology, #2525) (35).

***Quantitative real-time PCR analysis***

The TRIzol Reagent (Invitrogen, Paisley, UK) was utilized to extract total RNA. Total RNA concentrations of samples were quantified using spectrophotometry and after that carefully modified to a concentration of 0.5 μg/ml. Finally, cDNAs were reverse-transcribed from total RNA in a total amount of 20 μl comprising 5 μg total extracted RNA via a commercial kit (Thermo Scientific, Waltham, MA, USA). The sequences of the primers used are shown in Table1. 

The β-actin was employed as inner control. Quantitative real-time PCRs were performed in 48-well plates within capacities of 20 μl comprising 1 μl cDNA, 2 μl of the mix of reverse and forward primers, 7 μl deionized water, and 10 μl SYBER GREEN PCR master mix. One-step RT-PCR was performed in the Applied Biosystems 7500 Fast Real-Time PCR System (Applied Biosystems Deutschland GmbH, Darmstadt, Germany). We processed terms of cycling and melting in the following ways: one cycle for 10 min at 95 °C, followed by 40 cycles for 15 sec at 95 °C, for 30 sec at 58 °C, and 30 sec at 72 °C, and a last extension step (melt curve step) for 15 sec at 95 °C, for 60 sec at 60 °C, and 15 sec at 95 °C. Finally, quantitation was assessed through the Pfaffl procedure expressed as ratios (2^–∆∆Ct^ target:2^– ∆∆Ct^ reference) (36).

***Testicular oxidative stress***

The lower part of the frozen left testis was used for evaluation of testicular oxidative stress. The lipid peroxidation levels were measured and shown by the amount of malondialdehyde (MDA). To provide a solution of TBA-TCA-HCL, 375 mg of thiobarbituric acid (TBA) was solved in 2 ml of hydrochloric acid (HCL) and added to 100 ml of 15% TCA. 50 °C water bathing was utilized for the complete dissolution of sediment. The weight of tissue was measured, then it was homogenized with a potassium chloride 5.1% solution to get a 10% homogenized mix. Afterward, 1 ml of the homogenized mixture was mixed in 2 ml of the TBA-TCA-HCL solution and heated for 45 min in boiling water. After it cooled down, it was centrifuged for 10 min at 3500 rpm. The absorption was read using a spectrophotometer at 535 nm and finally, MDA content was presented as nmol/mg tissue protein. Superoxide dismutase (SOD), catalase (CAT) and Glutathione peroxidase (GPx) activities in the testis were assayed in accordance with the protocols of the kits used (Zellbio GmbH kits, Germany) (37). 

***Apoptosis assay***

Five-micrometer slides were deparaffinized and rehydrated. After that, they was rinsed three times by a nuclease-free phosphate buffer. POD *in situ* cell death detection kit (Roche, Germany) was used for identification of apoptotic cells in seminiferous tubules. All testicular tissue slides were pretreated via 0.3% H_2_O_2_ in methanol for 30 min at 25 °C. Before the enzymatic labeling, the slides were incubated in PBS with 20 μg/ml proteinase K (Roche, Germany) at 37 °C for 15 min. The sections were treated in a damp and dark chamber by 50 μl of TUNEL reaction mixture at 37 °C for 60 min, then the sections were hybridized in POD for 30 min and dyed by 3-3’-diaminobenzidine (DAB) for 15 min. In the final step, slides were counterstained by hematoxylin. Apoptotic cells were identified under a light microscope filtered by blue light as dark brown nucleus. Tagged germ cells were investigated for each rat in 20 tubules and cellular apoptotic index was demonstrated as the percent of tubules that had at least a TUNEL-positive cell. Also, the percent of TUNEL-positive cells in 100 tubules was demonstrated as tubular apoptotic index (38).

***Evaluation of sperm parameters***

The epididymis of each rat was removed from the right testis and minced in 5 ml Ham’s F10 medium and then put in the incubator at 37 °C with 5% CO_2_ for 30 min and removed 100 μl of this solution and dissolved in 900 μl Ham’s F10. A drop of the new solution was blended completely and added to the Neubauer chamber. We conducted the sperm count based on the standard protocol in 5 squares of 0.1 cm^2^ each, excluding the central zone. The total count was multiplied via a correction factor, 5×10^6^ (39-40). 

***Statistical analysis***

Data were analyzed via the SPSS version 20 statistical software package (IBM Company, SPSS Inc.). All findings were displayed as Mean±SEM. One-way ANOVA followed by Tukey’s *post hoc* analysis was conducted for all variables. A *P*-value of lower than 0.05 was considered significant. 

## Results

***Testicular oxidative stress***

**Figure 1:**
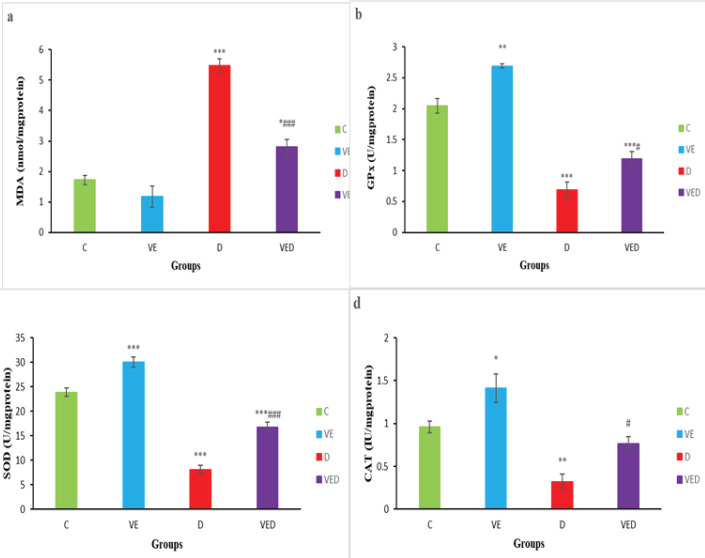
Testicular (a) malondialdehyde (MDA) content (nmol/mg protein), (b) Glutathione peroxidase (GPX) activity (U/mg protein), (c) Superoxide dismutase (SOD) activity (U/mg protein) and (d) catalase activity (IU/mg protein) of control (C), voluntary exercise (VE), diabetic (D) and voluntary exercise diabetic (VED) groups. Data are presented as Mean±SEM (n=8). Comparisons were done by one-way ANOVA followed by Tukey’s post hoc test. Statistical differences between control and different groups: *; *P*<0.05, **; *P*<0.01, ***; *P*<0.001, statistical differences between diabetic and different groups: #; *P*<0.05, ###; *P*<0.001

The findings indicated that MDA concentration was increased in D and VED groups compared with controls (*P*<0.001 to *P*<0.05). Voluntary exercise in the VED group significantly reduced the MDA levels in comparison with the D group (*P*<0.001, Figure 1a).

The findings revealed that the levels of testicular GPx, SOD, and catalase activities in the D group were notably lesser than controls (*P*<0.001 to *P*<0.01). The levels of GPx, SOD, and catalase activities in the VED group increased considerably compared with the D group (*P*<0.001 to *P*<0.05), however, the levels of GPx and SOD activities in the VED group were notably lower than controls (*P*<0.001, Figures 1b, c, and d). 

***miR-34a/SIRT1/p53 signaling pathway***

**Figure 2:**
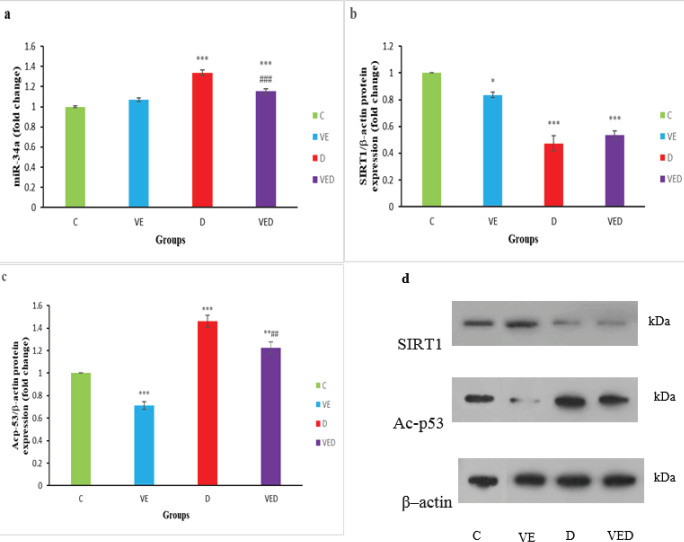
Testicular expression of miR-34a (a) and protein levels of Silent information regulator 1 (SIRT1) (b) and Ac-p53 (c) and their immunoblotting in control (C), voluntary exercise (VE), diabetic (D), and voluntary exercise diabetic (VED) groups. Data are presented as Mean±SEM (n= 8). Comparisons were done by one-way ANOVA followed by Tukey’s post hoc test. Statistical differences between control and different groups: *; *P*<0.05, **; *P*<0.01, ***; *P*<0.001, statistical differences between diabetic and different groups: ##; *P*<0.01, ###; *P*<0.001

The findings indicated that testicular miR-34a expression raised in D and VED groups compared with controls (*P*<0.001), and voluntary exercise could reduce the expression of miR-34a in the VED group compared with diabetic rats (*P*<0.001, Figure 2a). SIRT1 protein expression in the testis of rats in the VE group as well as diabetic animals in D and VED groups remarkably reduced in comparison with controls (*P*<0.001 to *P*<0.05). There was no remarkable difference between testicular SIRT1 protein expression of D and VED groups (Figure 2b). There was significant elevation of acetylated p53 protein expression in diabetic rats in D and VED groups compared with controls (*P*<0.001 to *P*<0.01), however, voluntary exercise in the VE group could diminish the acetylated p53 protein expression in comparison with controls (*P*<0.001). The acetylated p53 protein expression in the VED group was notably lower than the D group (*P*<0.01, Figure 2c).

***Apoptosis assay***

**Figure 3:**
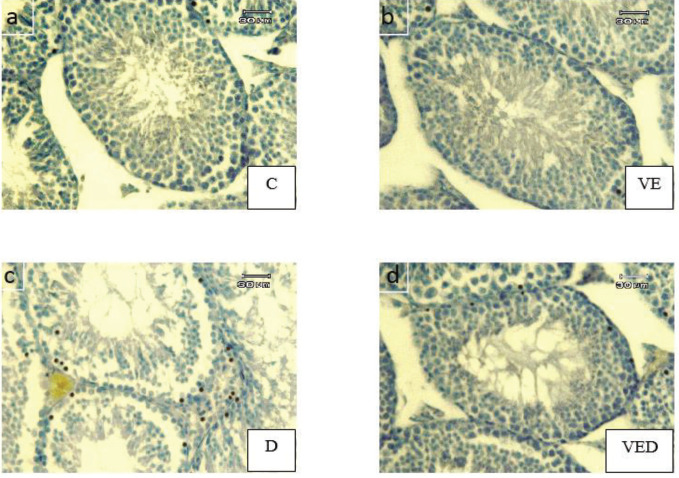
Photomicrographs of TUNEL test in the testicles of control (C), voluntary exercise (VE), diabetic (D), and voluntary exercise diabetic (VED) groups. The TUNEL-positive cells (apoptotic) display dark nuclei and TUNEL-negative cells (normal) display blue nuclei. (TUNEL immunohistochemistry staining, ×400)

**Figure 4:**
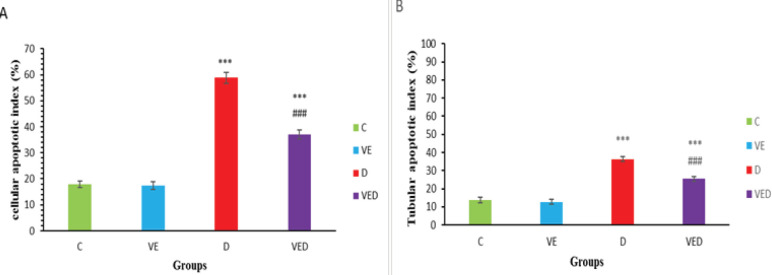
(A) cellular apoptosis index (%) and (B) tubular apoptosis index (%) in control (C), voluntary exercise (VE), diabetic (D) and voluntary exercise diabetic (VED) groups. Data are presented as Mean±SEM (n= 8). Comparisons were done by one-way ANOVA followed by Tukey’s post hoc test. Statistical differences between control and different groups: ***; *P*<0.001, statistical differences between diabetic and different groups: ###;* P*<0.001

As shown in Figures 3 and 4, the cellular apoptotic index, the percent of tubules that have at least one TUNEL-positive cell, was increased in D and VED groups in comparison with controls (*P*<0.001). There was remarkable decline in the VED group compared with the D group (*P*<0.001). Moreover, tubular apoptotic index, the percent of TUNEL-positive cells per one hundred tubules, was remarkably enhanced in D and VED groups in comparison with controls (*P*<0.001). Voluntary exercise in the VED group caused a notable decrease against the D group (*P*<0.001).

***The sperm parameters***

The findings showed that diabetes could reduce the total count, normal morphology, and viability of sperms compared with controls (*P*<0.001). Voluntary exercise in the VED group notably enhanced all parameters in comparison with the D group (*P*<0.001), however, sperm viability and normal morphology in this group were considerably lower than in the C group (*P*<0.001). The sperm motility in D and VED groups decreased remarkably compared with controls (*P*<0.001), however, the voluntary exercise caused an increment in this parameter compared with the diabetic group (*P*<0.001). Moreover, diabetes considerably enhanced the percentage of immotile sperm and diminished the percentage of fast, slow, and non-progressive sperms in the D group in comparison with controls (*P*<0.001). All sperm motility grades improved in the VED group compared with the D group, however, the fast, non-progressive, and immotile sperms in the VED group were remarkably different from those of controls (*P*<0.001, Tables 2 and 3).

**Table 1:** The sequences of primers utilized for the expressions of genes

**Gene**	**Forward primer **	**Reverse primer**
β-Actin	5՛-AAATCTGGCACCACACCTTC -3´	5՛-CCATCTCTTGCTCGAAGTCC -3´
SIRT1	5´-GTGAGAAAATGCTGGCCTAA -3´	5´-CTGCCACAGGAACTAGAGGA -3´
P53	5´-TGCTGAGTATCTGGACGACA -3´	5´-AAACACGAACCTCAAAGCTG -3´

**Table 2:** Sperm parameters in control (C), voluntary exercise (VE), diabetic (D), and voluntary exercise diabetic (VED) groups

	**c**	**ve**	**d**	**ved**
**Total count (×10** ^6^ **)**	44.80±3.421	44.80±2.775	24.40±3.362***	39.60±1.673###
**Viability (%)**	80.20±2.387	81.60±1.517	41.00±2.121***	61.60±2.408***###
**Motility (%)**	73.20±3.033	75.20±2.168	33.20±1.924***	53.60±1.517***###
**Normal morphology (%)**	71.40±2.608	74.40±2.608	34.40±2.966***	55.60±3.782***###

Data are presented as Mean±SEM (n=8). Comparisons were done by one-way ANOVA followed by Tukey’s post hoc test. Statistical differences between control and different groups: ***; *P*<0.001, statistical differences between diabetic and different groups: ###; *P*<0.001

**Table 3:** The sperm motility grade (%) in control (C), voluntary exercise (VE), diabetic (D), and voluntary exercise diabetic (VED) groups

	**C**	**vE**	**D**	**vED**
**A (** **fast progressive** **)**	42.60±2.510	42.60±4.219	21.80±3.347***	32.20±2.775***###
**B (** **slow progressive** **)**	11.20±2.588	11.60±1.673	4.400±1.342***	11.00±1.581###
**C (** **non-progressive** **)**	13.60±3.130	14.00±1.581	3.400±1.140***	6.000±2.000***
**D (** **motile in curved line** **)**	5.800±1.304	7.000±1.225	3.600±1.517	5.500±0.5774
**B+D**	17.00±2.915	18.60±2.074	8.000±1.732***	15.40±2.702###
**E ** **(immotile** **)**	26.80±3.033	24.80±2.168	66.80±1.924***	46.40±1.517***###

Data are presented as Mean±SEM (n=8). Comparisons were done by one-way ANOVA followed by Tukey’s post hoc test. Statistical differences between control and different groups: ***; *P*<0.001, statistical differences between diabetic and different groups: ###; *P*<0.001

## Discussion

This study explored whether voluntary exercise could improve reproductive complications of diabetes in the rat testicles. Male fertility deficiency and sexual dysfunction caused by diabetes are two important clinical complications with inadequate treatment options (41). 

We found that a high-fat diet-induced diabetes caused notable reduction in sperm parameters such as total count, normal morphology, motility, and viability. In the diabetic group, the number of fast, slow, and non-progressive sperms significantly decreased, although the number of immotile sperm significantly increased. These results are in line with early reports (42-44). Voluntary exercise improves sperm parameters in male diabetic rats probably through up-regulating the seminal antioxidant system and attenuating apoptotic factors (28, 45). 

The seminal plasma fructose is the main energy source for sperm motility and viability (46). In diabetic rats, seminal plasma fructose is increased because of sperm count reduction, which may prevent the use of fructose due to oxidative stress (46). A study in 2012 suggested that moderate exercise can cause a more appropriate environment for spermatogenesis (43), although a study in 2006 showed that exhaustive endurance exercise could reduce sperm concentration (47). As the voluntary exercise facility was prepared for rats in this study, this protocol is considered moderate intensity physical activity (48).

Our biochemical findings indicated that high-fat diet-induced diabetes caused a reduction in antioxidant enzyme activities. Previous research demonstrated the altered antioxidant pool and increased oxidative stress in the testicles of diabetic animals (49, 50). Performing exercises can selectively activate antioxidant enzymes based on the oxidative stress applied to particular tissues and the intrinsic antioxidant defense capacity (51). There is contradictory information on the correlation between variations in antioxidant enzyme activities and exercise (51). Previous studies have reported that the testes have the greatest cellular level of SOD (52, 53), which may be associated with the catalytic dismutation of superoxide anion (O_2_^−^) to hydrogen peroxide (H_2_O_2_) (54). Superoxide is the main reactive oxygen species (ROS) produced by spermatozoa (55). In normal physiological conditions, spermatozoa produce O_2_^−^ in smaller quantities for acrosomal reaction and capacitation (56). During disease states, this physiological production of ROS is declined which leads to an increment in testicular O_2_^−^ levels with an adverse effect on men fertility (49, 55). 

In this investigation, the biochemical results demonstrated that voluntary exercise diminished oxidative stress but increased the levels of GPx, CAT, and SOD enzymes in the testes of HFD-induced diabetic rats. Many investigations have reported that prolonged exercise positively changes oxidative homeostasis in cells and tissues by reducing the basal oxidative injury levels and enhancing resistance against oxidative stress (56-60). Also, regular exercise can lead to antioxidant capacity adaptation and protect cells from the adverse impacts of oxidative stress, which inhibits cellular damage (57).

MiRNAs can control the expression of genes through binding and modulating the translation of particular mRNAs (61). Many miRNAs in mammals are still waiting to be identified, and only a little is identified about the levels of miRNA expression or patterns in spermatogenesis. Therefore, it is necessary to study miRNA relating to spermatogenesis such as miR-34a. SIRT1 was proven to attach miR-34a back to p53. SIRT1 is an NAD^+^-dependent deacetylase, which suppresses the activity of p53 through post-transcriptional deacetylation of p53 protein (62). MiR-34a targets Sirt1 mRNA, resulting in a decline of SIRT1 protein (14), the impact of which reduces the deacetylase activity of SIRT1’s histone and elevated acetylated P53, as an activated form of the P53 protein (14, 63). The activation of P53 increases the transcription of the miR-34a gene, providing more miR-34a that reduces SIRT1 protein in testicles (24). Apoptosis is a physiological mechanism whereby unwanted or damaged cells are removed from the organism. Recent investigations revealed that miR-34a ectopic expression can induce apoptosis in cell lines of neuroblastoma (64). The pro-apoptotic roles of miR-34a were confirmed by a lot of studies in different cancer entities and some anti-apoptotic genes verified as miR-34 targets (20, 65, 66). MiR-34a inhibition protects cells in wild-type p53-expressing cells from the DNA damage that induces apoptosis, showing that miR-34a is needed for p53 induced apoptosis (65). 

In this study, our data revealed that testicular miR-34a and Ac-p53 expression increased in testes of diabetic rats. Our results were in line with Jiao *et al.* study which showed miR-34a increased in the testis of diabetic mice (24). Another study indicated that p53 activation was augmented in the testicles of diabetic mice (67). Moreover, reduced SIRT1 protein was detected in the testicles of diabetic rats (68). Supporting these results, the present study showed reduced testicular SIRT1 expression in diabetic rats. This elevated miR-34a level might be responsible for the reduced expression of SIRT1 protein under the diabetic condition. Moreover, the TUNEL method findings indicated the great number of TUNEL-positive apoptotic cells as well as the elevated cellular and tubular apoptotic indexes in testicles of diabetic animals. These results were in line with former research (69). Treatment with voluntary exercise increased SIRT1 expression in testicular tissue and reduced miR-34a and Ac-p53 expression in diabetic rats. Voluntary exercise also reduced the number of TUNEL-positive apoptotic cells in testicles of diabetic animals and could reduce the cellular and tubular apoptotic indexes in VED animals.

## Conclusion

The findings disclosed that voluntary exercise can be an effective method for improving spermatogenesis and reducing apoptosis in testicles of diabetics by reducing oxidative stress and alteration in the miR-34a/SIRT1/p53 pathway. However, many investigations are necessary to clarify these results.
